# S-Net: a multiple cross aggregation convolutional architecture for automatic segmentation of small/thin structures for cardiovascular applications

**DOI:** 10.3389/fphys.2023.1209659

**Published:** 2023-11-02

**Authors:** Nan Mu, Zonghan Lyu, Mostafa Rezaeitaleshmahalleh, Cassie Bonifas, Jordan Gosnell, Marcus Haw, Joseph Vettukattil, Jingfeng Jiang

**Affiliations:** ^1^ Department of Biomedical Engineering, Michigan Technological University, Houghton, MI, United States; ^2^ Center for Biocomputing and Digital Health, Health Research Institute, Institute of Computing and Cybernetics, Michigan Technological University, Houghton, MI, United States; ^3^ Betz Congenital Health Center, Helen DeVos Children’s Hospital, Grand Rapids, MI, United States

**Keywords:** automatic segmentation, cardiac wall, intracranial aneurysm (IA), small/thin structure, fully convolutional network (FCN)

## Abstract

With the success of U-Net or its variants in automatic medical image segmentation, building a fully convolutional network (FCN) based on an encoder-decoder structure has become an effective end-to-end learning approach. However, the intrinsic property of FCNs is that as the encoder deepens, higher-level features are learned, and the receptive field size of the network increases, which results in unsatisfactory performance for detecting low-level small/thin structures such as atrial walls and small arteries. To address this issue, we propose to keep the different encoding layer features at their original sizes to constrain the receptive field from increasing as the network goes deeper. Accordingly, we develop a novel S-shaped multiple cross-aggregation segmentation architecture named S-Net, which has two branches in the encoding stage, i.e., a resampling branch to capture low-level fine-grained details and thin/small structures and a downsampling branch to learn high-level discriminative knowledge. In particular, these two branches learn complementary features by residual cross-aggregation; the fusion of the complementary features from different decoding layers can be effectively accomplished through lateral connections. Meanwhile, we perform supervised prediction at all decoding layers to incorporate coarse-level features with high semantic meaning and fine-level features with high localization capability to detect multi-scale structures, especially for small/thin volumes fully. To validate the effectiveness of our S-Net, we conducted extensive experiments on the segmentation of cardiac wall and intracranial aneurysm (IA) vasculature, and quantitative and qualitative evaluations demonstrated the superior performance of our method for predicting small/thin structures in medical images.

## 1 Introduction

Cardiovascular disease is one of the leading causes of death, accounting for one death every 34 s in the United States^1^. A comprehensive understanding of cardiac function and brain vessel integrity is essential for preventing, diagnosing, and treating this life-threatening disease. Automatic medical image segmentation of cardiac imaging data plays a crucial role in medical 3D printing (Lindquist et al., 2021), computer modeling (Jou et al., 2003; Cebral et al., 2005), and computer-aided diagnosis of cardiovascular systems (Lindquist Liljeqvist et al., 2021; Sunderland et al., 2021; Rezaeitaleshmahalleh et al., 2023c), assisting cardiologists, radiologists, and surgeons in making clinical decisions efficiently.

In the last 2 decades, considerable research efforts have been devoted to computational hemodynamics (Jou et al., 2003; Cebral et al., 2005). Accurate “patient-specific” vasculature segmentation from 3D imaging data is critical for subsequent numerical evaluations of each patient’s hemodynamic environment. However, an automated workflow for model creation in computational hemodynamics still requires considerable attention (Mu et al., 2023a; Rezaeitaleshmahalleh et al., 2023b; Lyu et al., 2023), particularly in delineating small brain arteries (approximately 0.5 mm in diameter). Similarly, although considerable research has been devoted to segmenting cardiac imaging data (Suri, 2000; Petitjean and Dacher, 2011), whole heart wall automatic segmentation remains challenging because the thickness of the atrial wall is particularly thin (approximately 1–4 mm, and down to 0.5 mm in pediatric populations). Existing studies have only focused on myocardial wall segmentation of the left and right ventricles (Zhu et al., 2013; Yang et al., 2018). Those unmet needs motivate the work presented in this study.

Early medical image segmentation methods include active contours (Xu and Prince, 1998), template matching (Lalonde et al., 2001), edge detection (Zhao et al., 2006), shape modeling (Tsai et al., 2003), machine learning (Zhang et al., 2004), etc. More recently, deep-learning-based methods have been developed to extract abundant and powerful data-specific features from cardiac and brain imaging data. Typically, most CNN models developed for medical image segmentation are of the encoder-decoder type, which is one of the most popular end-to-end architectures, e.g., fully convolutional network (FCN) (Yuan et al., 2017), U-Net (Ronneberger et al., 2015), and their variants (Bhalerao and Thakur, 2019; Isensee et al., 2021). Among these structures, the encoder is usually deployed to progressively extract higher-level medical image features. At the same time, the decoder is generally employed for recovering and integrating the extracted (multi-scale) features back to the original image size. Eventually, this end-to-end configuration generates the final segmentation result. Theoretically, as the network goes deeper, more high-level features are extracted at the expense of losing low-level detail information. Although skip connections generally help propagate local features from the encoder to the decoder, they still fail to adequately capture small/thin anatomical structures in cardiovascular application. This shortcoming is well noted in the literature (Mu et al., 2023a).

**FIGURE 1:**
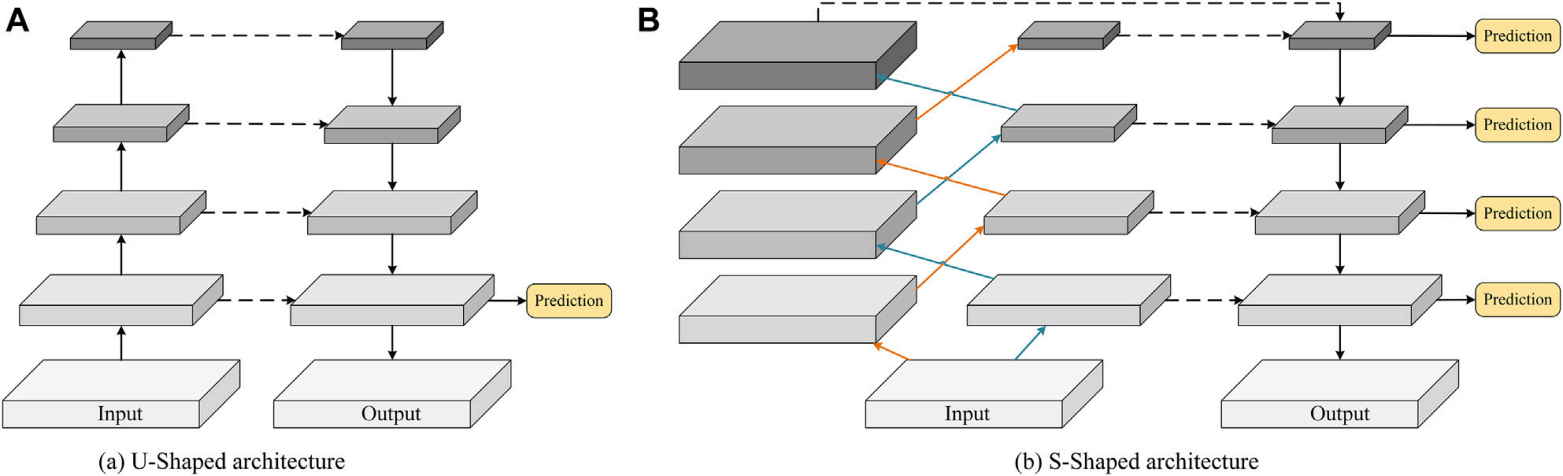
Schematic comparison of **(A)** the conventional U-shaped structure and **(B)** the proposed S-shaped structure.

Currently, the U-shaped architecture in the classic U-Net model (Ronneberger et al., 2015) achieves satisfactory performance in segmenting large structures but suffers from significant limitations. Recall that the U-shaped architecture contains both top-down and bottom-up paths (see Figure 1A). First, a large amount of spatial detail information is lost during the downsampling of the bottom-up path and cannot be easily recovered. Second, the top-down path may gradually weaken the global context information in high-level features, resulting in incomplete segmentation results. Third, making predictions only for high-resolution feature maps with weaker semantics inevitably limits the expressiveness of small/thin object recognition. Existing approaches address these problems by introducing an attention mechanism (Mu et al., 2023a) or self-attention (Valanarasu et al., 2021a) into the U-shaped structures, refining the feature map in a recursive (Alom et al., 2019) or cascading (Mu et al., 2023b) manner, etc. However, the abovementioned newer networks are configured to focus only on high-level features and cannot detect some low-level detail structures.

In this paper, we investigate an alternative approach, i.e., controlling the receptive field size of the convolutional filters in the encoder. Thus, the new configuration can effectively guide a CNN model to capture fine-grained details and small/thin structures even in deeper layers. Specifically, we discard the traditional U-shaped framework and propose constructing two resampling and downsampling branches with an S-shaped cross aggregation to control receptive field size. As a result, we name our new network configuration S-Net, which enables learning of low-level detail information and high-level discriminative knowledge in the encoding stage. As shown in Figure 1B, compared with the bottom-up encoder (see Figure 1A), the proposed S-shaped network adds resampling layers to the encoder to avoid spatial information loss while ensuring a small receptive field for capturing low-level details during convolution.

**FIGURE 2:**
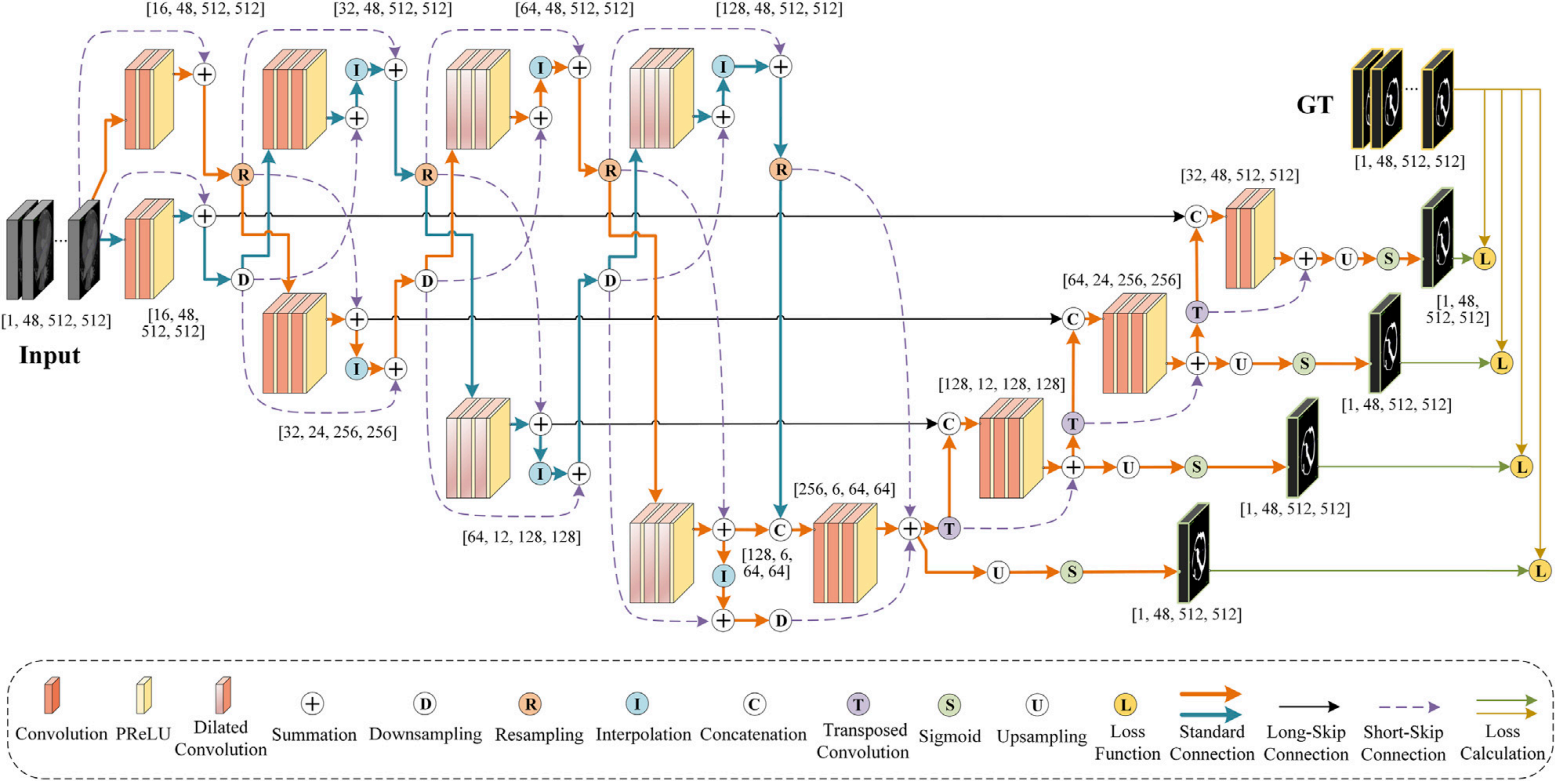
An illustrative diagram showing the architecture of the proposed S-Net.

Furthermore, considering the characteristics and complementarity of downsampling and resampling branches in our S-Net, we design a simple multiple cross aggregation module (MCAM) to efficiently integrate multilevel high-level and low-level features efficiently, ensuring robust and comprehensive feature representation. In particular, we also configure dilated convolutions of different specifications in the encoder to extract rich global context information and propagate it to the top-down decoder through lateral connections to strengthen the global dependence of decoding features. Last, we perform supervised prediction of features on all decoding layers, which combines semantically strong, low-resolution features with semantically weak, high-resolution features along the top-down path. The new S-Net design is anticipated to enhance the segmentation of large and small anatomical targets, and its architecture is illustrated in Figure 2.

In this study, we perform segmentation experiments of the heart wall and intracranial aneurysm (IA) vasculature to demonstrate the merits of the proposed S-Net. The whole heart wall segmentation requires dealing with thin structures, while segmenting brain vasculature with IAs involves small vessel annotations. The success of both applications is highly dependent on the ability of CNNs to discriminate fine-grained structures. The performance of the proposed S-Net is compared with five other state-of-the-art medical segmentation models.

Overall, our contributions are summarized as follows. First, we propose an efficient dual-branch encoder to explore spatial details and global contextual information. Moreover, we design a multiple cross-aggregation module to exploit the complementarity of these two branches for improving the feature representation capability on small structures. Second, we improve the learning effectiveness of S-Net by optimizing semantic and localization knowledge based on top-down multilevel supervision. Third, we experimentally explore the proposed S-Net for image segmentation of the heart wall and IA vasculature, demonstrating that our model achieves state-of-the-art performance both quantitatively and qualitatively, especially for small volume prediction.

## 2 Related works

This section briefly reviews U-Net-based medical image segmentation architectures and cardiac wall segmentation models.

### 2.1 U-Net architecture

Since its proposal, U-Net (Ronneberger et al., 2015), with an encoder-decoder structure, has inspired substantial further developments. Its variants have become widely adopted tools for medical image segmentation tasks. By extending the dimensionality of the basic U-Net framework, 3D U-Net (Çiçek et al., 2016) enables 3D volume segmentation, which has been of significant effect in many biomedical applications. Some attention-based U-Nets (Oktay et al., 2018; Mu et al., 2023a) emphasize local information by employing attention units to allow the network to focus on specific objects of importance while ignoring unnecessary regions. To segment targets of various sizes and shapes, inception U-Nets (Chen et al., 2018; Zhang et al., 2020) utilize convolutional filters of multiple sizes in the same network layer to analyze images with different salient regions efficiently. To overcome the training difficulty of convergence caused by the degradation of deep CNN features, residual U-Nets (Bhalerao and Thakur, 2019; Yu et al., 2019) acquire feature maps from one network layer and add them to a deeper layer to improve the performance. In addition, recurrent U-Nets (Alom et al., 2019; Jiang et al., 2020) optimize the expressiveness of the feature maps by merging recurrent feedback loops into the convolutional layer to obtain the context of adjacent units. To compensate for the information loss in the deeper layers of CNNs, dense U-Nets (Li et al., 2018; Wang et al., 2019) construct identity mappings for each layer, which depend not only on the former layer but also on all previous layers.

In summary, those U-Net variants have been designed to optimize the segmentation results by tuning the network structure, introducing new modules, etc. However, those variants largely follow the encoder-decoder design constructed by downsampling and upsampling convolutional layers (See Figure 1A). Recall that the existing U-shaped configuration is prone to spatial information loss and insufficient knowledge acquisition of small/thin targets. Thus, in this study, our work is fundamentally different than that of prior publications.

### 2.2 Cardiac wall segmentation

Cardiac wall segmentation faces two main challenges. First, the mixture of contrast, blood, and dynamic myocardial structures causes blurred boundaries of the heart wall, making them difficult to be distinguished. Second, the atrial wall, interatrial septum, and cardiac valves are thin and irregularly shaped, which makes them highly unrecognizable. Although there have been a wealth of studies addressing segmentation of the whole heart (Zhuang et al., 2010; Xu et al., 2019), four chambers (Zheng et al., 2008), left and/or right ventricle (Ringenberg et al., 2014; Lu et al., 2019), and left and/or right atrium (Tobon-Gomez et al., 2013; Tobon-Gomez et al., 2015), there appear to be no studies of whole heart wall segmentation.

Notably, Zhu et al. proposed an automated method based on variational region growth to segment the myocardial walls of right and left ventricles using cardiac computed tomography (CT) images (Zhu et al., 2013). Yang et al. presented a multi-component deformable model combined with 2D-3D U-Net for segmenting the ventricular walls from cardiac magnetic resonance imaging (MRI) (Yang et al., 2018). Ye et al. applied a PC-UNet to segment the left ventricle myocardium wall in conjunction with CT data (Ye et al., 2021). However, none of the early research involved segmenting the whole heart wall. This is because the segmentation of the whole heart wall is challenging for the following reasons. First, the heart organ has multiple chambers and large vessels with complex geometry. The shape of the heart wall varies considerably between different subjects or in the same subject with different cardiac conditions, and this variation in shape is particularly pronounced when pathological conditions are involved. Therefore, it is difficult to accurately capture the complex shape of the whole heart wall using priori models trained from a limited training dataset. Second, depending on the intensity distribution (i.e., texture pattern) of the medical images, some boundaries between anatomical substructures are visually indistinct, e.g., the valve planes that separate the atria and great vessels from the ventricles, the boundaries between the left atrium and the pulmonary veins and between the right atrium and the superior/inferior vena cava, and the thin walls of the atria and vessels. These ill-defined boundaries make fully automated whole heart wall segmentation challenging to achieve. Segmenting thin cardiac structures like atrial walls is also a technically challenging problem. Third, the intensity distribution between some adjacent tissues or substructures is highly similar; e.g., the intensity of the myocardium is analogous to the neighboring papillary muscles, liver, and body muscles. Therefore, segmentation models relying only on image intensities have difficulty separating the heart wall from similar tissues. Finally, due to the complex motion within the heart, the imaging data may contain severe motion artifacts, interference noise, and intensity inhomogeneities, leading to unsmooth and undesirable delineation of the heart wall.

## 3 Proposed method

The overall pipeline of our S-Net is depicted in Figure 2. It consists of eight coding blocks, four decoding blocks, and four supervision layers, where every two coding blocks form a densely connected MCAM. In this section, we first describe the structure of our S-Net model. Later, we elaborate on the proposed MCAM, and finally, we present the specific implementation details.

### 3.1 S-Net architecture

The essence of the proposed S-Net is a fully convolutional network similar to the classic U-Net (Ronneberger et al., 2015), consisting of encoding and decoding, but unlike the symmetric U-shaped structure of U-Net, the information propagation path of our S-Net in the encoding stage is interleaved, approximately following an S shape. Specifically, two strategies are used at the encoding stage to encode CT scans’ small/thin structures. First, we use convolution and downsampling to acquire four-layer features by gradually halving 3D sizes and increasing the number of channels. Second, we construct four-layer features with increasing channel numbers, but the 3D sizes remain the same as the input image patch for perceiving small targets. At the decode stage, we perform multiple cross-aggregation on these encoded features: Long-skip connections are used to fuse them to complement the four-layer upsampled convolutional features. Hence, abundant structural information is decoded for considerable performance gains in delineating small/thin objects.

**FIGURE 3:**
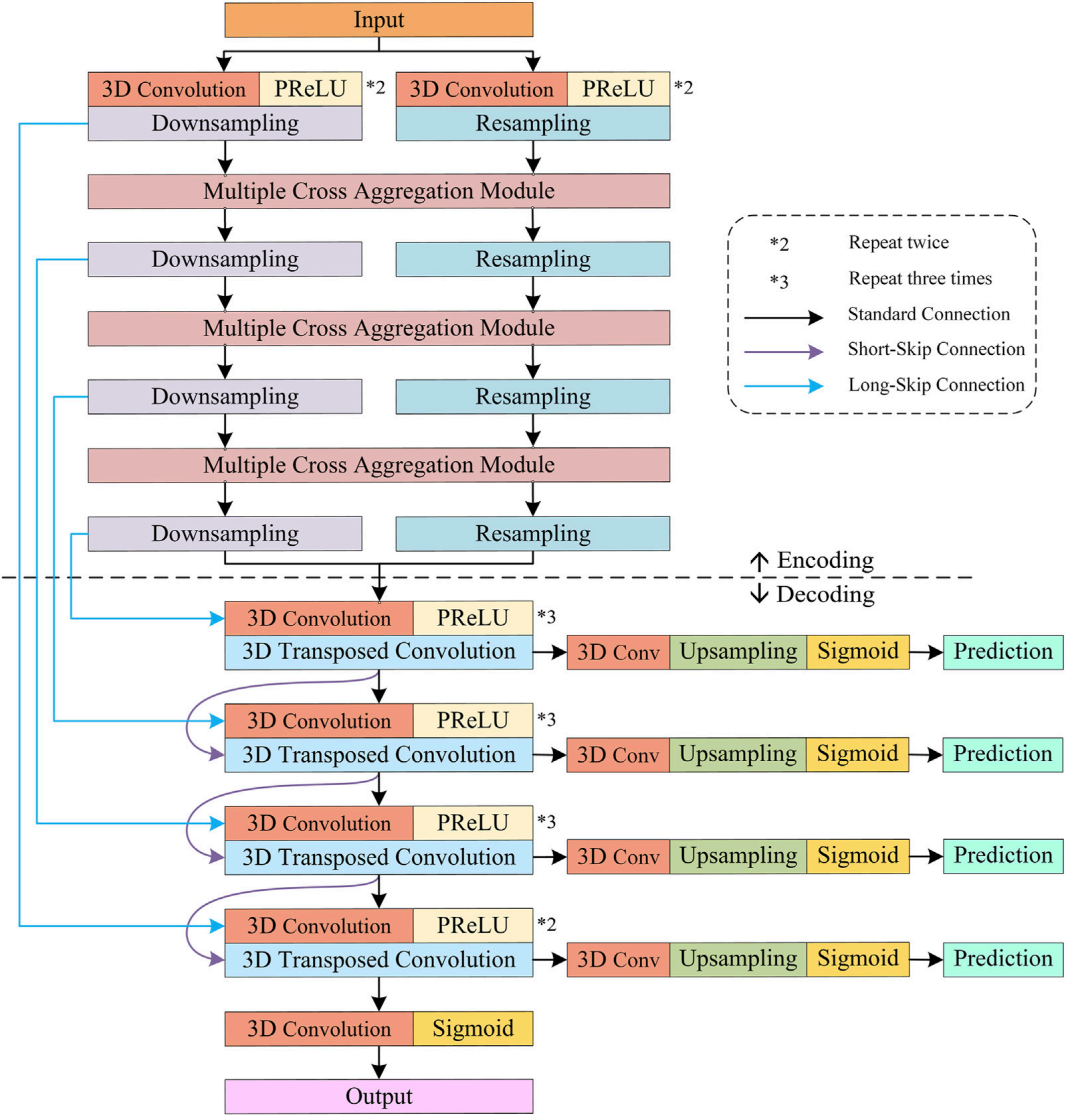
Encoding and decoding configurations of the proposed S-Net.

Furthermore, we leverage the Sigmoid activation function to implement layer-by-layer prediction on the multi-scale features generated by different decoding layers and calculate the errors between the predicted results and the ground truths. Errors are then back-propagated to update the model training parameters. The encoding and decoding structures of the proposed S-Net are illustrated in Figure 3.

#### 3.1.1 Rationale

Regarding the conventional U-Net and its variants, the feature size in the encoding stage is gradually decreasing. For an input 3D image of size D×W×H , after 3D convolution and 3D pooling operations, the size of the *i*th layer feature map is reduced to $\frac{D}{2^{i}}\times \frac{W}{2^{i}}\times \frac{H}{2^{i}}$ ; thus, for a 3D convolution with a kernel size of $3^{3}$ , its relative receptive field with respect to the features of the *i*th layer becomes ${\left.{\left(2\right.}^{i}\times 3\right)}^{3}$ . As a result, the receptive field of the convolutional layers in the encoder increases as the network deepens, making the deeper layers focus on high-level (semantic) features and, therefore, it cannot extract the knowledge for segmenting small/thin objects, fine details, etc. In light of this finding, we added a series of non-downsampled convolutional layers to the decoder, attempting to maintain a similar resolution to the input image at each layer, and interacting with the original downsampled convolutional layers to encode objects of different scales. Since the dimensions of the added convolutional layers are all kept as D×W×H , the $3^{3}$ convolution operations maintain the same small $3^{3}$ receptive field for each layer, enabling the network to perceive tiny structures.

#### 3.1.2 Encoder and decoder

**Encoder.** Dedicated to fine-grained 3D segmentation, the encoder of the proposed S-Net contains two main branches, i.e., a downsampled branch of four convolutional blocks with decreasing resolution and a resampled branch of four convolutional blocks preserving the original resolution. In particular, each convolution block includes two or three 3D convolutions followed by a parametric rectification linear unit (PReLU) activation function. Similarly, the channel numbers in the convolutional blocks of both branches increase as the network goes deeper. In addition, the first two convolution blocks have a kernel size of $3^{3}$ , with a stride of 1 and a padding of 1. The last two convolution blocks are mainly based on four different dilated convolutions with kernel size $3^{3}$ and stride 1, but with padding and dilation of {2, 2}, {3, 3}, {4, 4}, and {5, 5}, respectively, and their corresponding receptive fields are $3^{3}$ , $7^{3}$ , $9^{3}$ , and $11^{3}$ . Such a convolution setting undoubtedly allows the encoder to have a diverse range of receptive fields, which helps capture multi-scale targets.

Moreover, it is important to mention that for the two encoding branches, the downsampling relies on a 3D convolution with a kernel size of $2^{3}$ and a stride of 2 for halving the 3D feature size, while the resampling is based on a 3D convolution with a kernel size of $3^{3}$ , a stride of 1, and a padding of 1 to retain the original 3D size. More importantly, the two encoding branches interact with information through MCAM to learn multi-scale objective knowledge and propagate discriminative information from the encoding layer to the decoding layer of the identical resolution through long-skip connections, contributing to recovering the lost spatial information from downsampling encoding.

**Decoder.** As shown in Figure 3, the decoder comprises four convolution blocks with doubled resolution and halved channel numbers, each containing two to three 3D convolutions, followed by PReLU. Specifically, all the 3D convolutions have a kernel size of $3^{3}$ , a stride of 1, and a padding of 1. Also, all four 3D transposed convolution operators have a kernel size of $2^{3}$ and a stride of 2 to progressively recover the full spatial resolution of the network output. Subsequently, the feature maps generated by transposition convolution are 1) concatenated with the feature maps in the encoding path by long-skip connections to integrate more accurate pixel localization information and 2) provided with high-level semantic information for the next layer through short-skip connections (He et al., 2016). In the last layer, a 3D convolution with a kernel size of 1×1×1 is added to reduce the channel number of the output image to the label number, and the result is forwarded to the Sigmoid activation function to obtain the predicted voxels.

#### 3.1.3 Loss function

Typically, the semantic information of higher-level features in the decoder is gradually diluted along the top-down path; thus, there are significant semantic gaps in the feature maps generated by multiple convolutional layers of different depths. Generally, global contextual information is gradually ignored as the feature resolution of additional decoding layers increases. Therefore, the traditional U-Net structure using only high-resolution features with weak semantics for prediction will inevitably omit small/thin targets. Given this, the proposed S-Net enriches the semantic and location information by predicting the features of different decoding layers separately, thus enhancing the representation ability for small/thin object segmentation.

Specifically, as shown in Figure 3, for the features generated by the four decoding blocks, a 1×1×1 3 D convolution is first applied to reduce the channel number of these features to 1. The feature size is adjusted to the original image size by trilinear upsampling. Finally, the prediction results are obtained by the Sigmoid activation function, which will be utilized to calculate the back-propagation errors for updating the model parameters.

Let $P_{i}^{0}$ and $P_{i}^{1}$ denote the predicted background and foreground voxels, respectively, where $i\in \left\{1,2,3,4\right\}$ indicates the predictions of the four decoding layers. Meanwhile, $G^{0}$ and $G^{1}$ denote the voxels of the two labels corresponding to the ground truth (GT), respectively. Correspondingly, the proposed loss function between the predictions and the ground truth is defined as follows:

$$L=\sum_{i=1}^{4}\frac{\left|G^{1}\cap P_{i}^{1}\right|}{\left|G^{1}\cap P_{i}^{1}\right|+\alpha \left|G^{0}\cap P_{i}^{1}\right|+\beta \left|G^{1}\cap P_{i}^{0}\right|}=\sum_{i=1}^{4}\frac{\text{TP}}{TP+\alpha \times FP+\beta \times FN}$$

where $\left|G^{1}\cap P_{i}^{1}\right|$ , $\left|G^{0}\cap P_{i}^{1}\right|$ , and $\left|G^{1}\cap P_{i}^{0}\right|$ indicate the True Positive (TP), False Positive (FP), and False Negative (FN), respectively. The hyperparameters α and β are exploited to control the trade-off between FPs and FNs. It is worth noting that in this paper, we set α to 0.3 and β to 0.7 to emphasize FN over FP, i.e., giving more weight to Recall ( $\frac{\text{TP}}{\text{TP}+\text{FN}}$ ) than to Precision ( $\frac{\text{TP}}{\text{TP}+\text{FP}}$ ). Focusing more on Recall than Precision increases the probability of false detection of non-heart walls as heart walls to some extent but avoids the probability of missing detection of true heart walls. We experimentally verified that the weighting configurations of 0.3 and 0.7 for FP and FN are most effective for trading off the miss detection and false detection rates. Such a setup avoids the missed detection of small targets to a certain extent and improves the generalization ability to imbalanced data during training.

### 3.2 Multiple cross aggregation module

**FIGURE 4:**
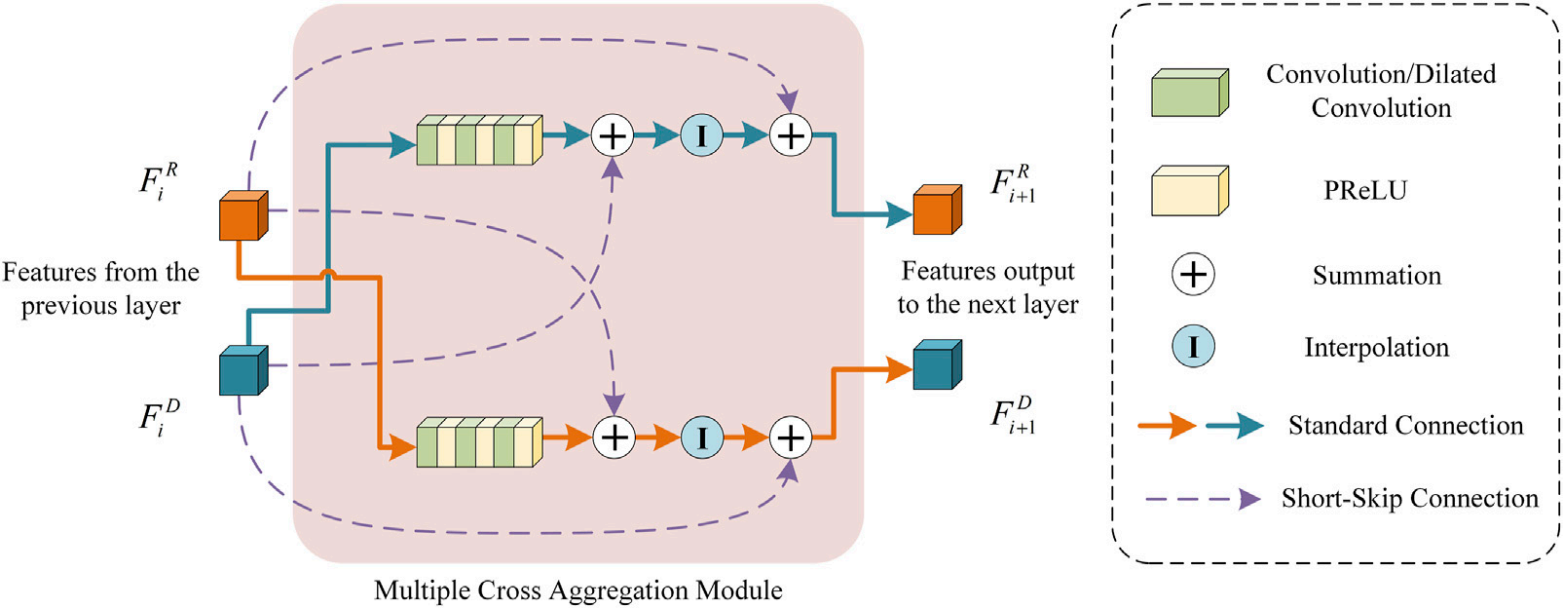
An Illustration of the proposed multiple cross-aggregation module.

To fully exploit the feature representation capability of the down-sampling and resampling branches in the encoder, we propose a multiple cross-aggregation module to integrate the features of both branches on each encoding layer. Since the feature scales of the two branches are different and the encoded discriminative information also differs, we attempt to capture complementary features from the two branches to further improve the quality of the features learned by the deep network. The structural details of the proposed multiple cross-aggregation module can be seen in Figure 4.

As shown in Figure 4, let the feature maps from the downsampling and resampling branches be denoted as $F_{i}^{D}$ and $F_{i}^{R}$ , respectively, where $i\in \left\{1,2,3,4\right\}$ represents the *i*th encoding layer. For the features $F_{i}^{D}$ and $F_{i}^{R}$ generated from the previous (i-1)^th^ layer, three 3D convolutions followed by PReLUs are first performed to extract deeper features. Next, the features before and after convolution are combined based on short-skip connections for facilitating network convergence. Then, trilinear interpolation is employed to upsample/downsample the convolutional features to a specific size (i.e., the feature size of the other branch) to obtain the cross-aggregated features. Those cross-aggregated features are finally added to features of the other branches through the residual short-skip connections to generate new complementary features $F_{i+1}^{R}$ and $F_{i+1}^{D}$ that are forwarded to the next layer.

Formally, the processing of multiple cross-aggregation can be expressed as:

$$F_{i+1}^{R}=F_{i}^{R}+Interp\left(F_{i}^{D}+PReLU\left(Conv\left(F_{i}^{D}\right)\right)\right),$$

$$F_{i+1}^{D}=F_{i}^{D}+\text{Interp}\left(F_{i}^{R}+\text{PReLU}\left(\text{Conv}\left(F_{i}^{R}\right)\right)\right),$$

where Conv,PReLU, and Interp denote the 3D convolution, PReLU, and trilinear interpolation operations, respectively. By extracting the complementary features from two branches of different scales, it will be beneficial to improve the network’s segmentation performance, making it capable of capturing fine-grained targets.

### 3.3 Implementation details

In this study, the proposed S-Net model is implemented by PyTorch framework (Version 2.1). To train and test the S-Net, we deploy patch-based learning by randomly cropping the original image along its vertical axis into a series of 48×512×512 voxel patches as the input to the network. In particular, the feature maps generated by the resampling branch in the encoder maintain the original size to cross-aggregate with the features whose size is gradually halved in the downsampling branch. Both training and testing tasks are accelerated by dual Tesla V100 PCIe GPUs with 32 GB memory. It is worth noting that when out-of-memory occurs during training, the feature size in the resampling branch will be appropriately scaled to fit the limited GPU RAM. For the specific setup, our model is trained for 1,000 epochs utilizing the Adam optimizer with a batch size of 2 and an initial learning rate of $1\times 10^{-4}$ . In addition, during training, a dropout operation is performed after each encoding and decoding layer to reduce overfitting; i.e., some elements of the output features are randomly zeroed with a probability of 0.3 using samples from the Bernoulli distribution.

## 4 Experiments

In this section, we first describe the experimental datasets and evaluation metrics used and then validate the superiority of the proposed S-Net through comparative experiments and ablation studies.

### 4.1 Experimental datasets

We have fully demonstrated the application of our S-Net in two different segmentation tasks. The first task was to segment the whole heart wall in the cardiac CT data (Zhuang and Shen, 2016) provided by the Multi-Modality Whole Heart Segmentation (MM-WHS) challenge^2^. We selected 40 CT images in NIFTI format having 512×512 pixels with 177–363 slices, and two experienced operators manually annotated the heart walls to obtain the GT labels. Specifically, we divided the data into 30 training images and ten testing images. The second task was to segment the IA and its vasculature in the IA dataset (Mu et al., 2023a), containing 23 3D rotational angiography (3DRA) images with a resolution of 256×256×256 . We used 15 cases for training and 8 cases for testing.

### 4.2 Evaluation metrics

We employed six widely used metrics to assess the performance of our S-Net and five other state-of-the-art deep-learning segmentation methods, including four volume measures, i.e., dice similarity coefficient (DICE), relative volume error (RVE), sensitivity, and specificity, and two surface measures in mm, i.e., 95% Hausdorff distance (HD95) and average symmetric surface distance (ASSD). A more detailed description of these evaluation metrics can be found in our previous publication (Mu et al., 2023a). All metrics were computed by comparing the predicted segmentation maps of all test data with their corresponding GTs. The mean and 95% confidence level of the calculated metrics are provided in the later experimental section.

### 4.3 Comparison results

To verify the effectiveness of the proposed S-Net, we compared it with five state-of-the-art 3D medical image segmentation models, including 3DUNet (Çiçek et al., 2016), SegNet (Badrinarayanan et al., 2017), 3DResUNet (Bhalerao and Thakur, 2019), KiU-Net (Valanarasu et al., 2021b), and nnUNet (Isensee et al., 2021). All algorithms are provided by respective authors of methods mentioned above, and for a fair comparison, we utilized the same experimental setup for training and testing. The average training time for our S-Net to complete each epoch was 1.227 min, while the other five comparison models consumed 0.697, 0.689, 0.855, 0.796, and 3.717 min, respectively.

**FIGURE 5:**
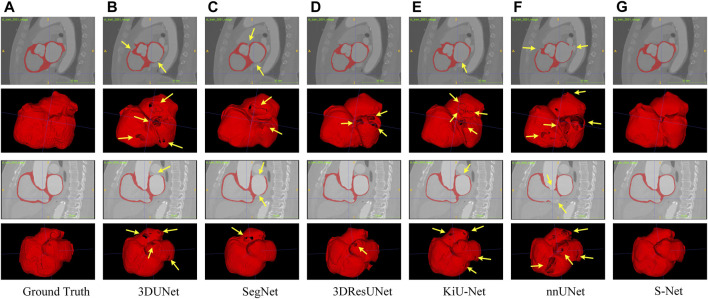
Qualitative comparison of six different models for segmenting the whole heart wall. Sagittal and 3D views are provided for each case. Arrows point to holes in the arterial wall. Images in column **(A)** are the Ground Truth and columns **(B–G)** represent the prediction results of 3DUNet, SegNet, 3DResUNet, KiU-Net, nnUNet, and our S-Net, respectively.

**FIGURE 6:**
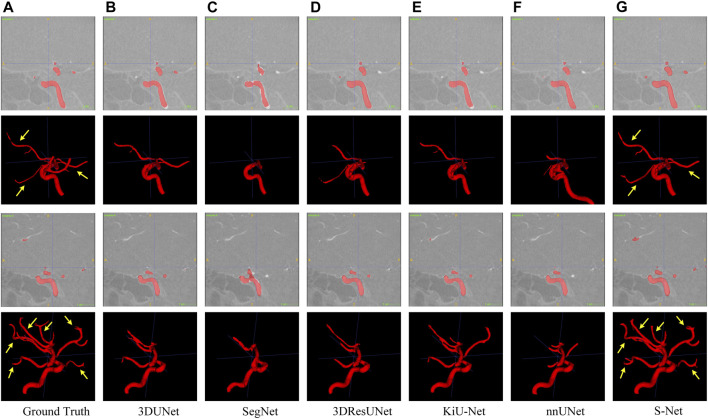
Qualitative comparison of six different models for segmenting the IA vasculature. Sagittal and 3D views are provided for each case. Arrows point to long segments of small vessels. Images in column **(A)** are the Ground Truth and columns **(B–G)** represent the prediction results of 3DUNet, SegNet, 3DResUNet, KiU-Net, nnUNet, and our S-Net, respectively.

**Qualitative Comparison.** To subjectively demonstrate the advantages of our S-Net, we provide some visual examples of the various models for heart wall and IA vasculature segmentation, as shown in Figure 5 and Figure 6. It can be easily seen that our model can generate more accurate and complete segmentation results than other compared methods. As observed in Figure 5, the surfaces of the heart walls segmented by the other five methods have large or small holes (indicated by the yellow arrows). Since quantitative assessment of cardiac function is primarily achieved by analyzing the shape attributes like heart wall thickness, enclosed area, or shape variation of the heart wall boundaries, it is crucial to completely and accurately determine the heart wall’s internal (endocardial) and external (epicardial) boundaries. Although other existing models have the ability to segment the heart wall, holes on the surface and internal mis-segmentation greatly affect the evaluation of cardiac function.

In contrast, our S-Net can segment the intact heart wall. These results (see Figure 5G) indicate that our model has a more robust characterization capability to capture the structural content and surface details, which greatly contribute to the subsequent analysis of cardiac function. The comparison example illustrated in Figure 6 shows that the other models perform well for segmenting large arteries (e.g., internal carotid artery) but are prone to miss some small vessels. This observation suggests that our S-Net is more effective in modeling long-range dependencies and small structures than other CNN models, achieving segmentation maps closest to the ground truth masks. Meanwhile, the multilayer supervision also drives the network to efficiently integrate semantic and localization knowledge, which is crucial for generating high-quality prediction results.

**Quantitative Comparison.** Table 1 and Table 2 list the quantitative results of the cardiac wall and IA vasculature segmentation in terms of six quantitative metrics. From Table 1, we can observe that the average scores of our S-Net for almost all metrics on the testing set outperform all state-of-the-art comparative models. In terms of DICE and Sensitivity scores, our S-Net achieved gains of 0.0631 and 0.0848 compared with the second-ranked nnUNet and 3DUNet, respectively. For HD95 and ASSD scores, the surface errors between our results and GT are reduced by 4.4573 mm and 0.7106 mm, respectively, compared with the suboptimal nnUNet. For the RVE and Specificity scores, we have only 0.0003 and 0.0005 differences compared with the first-ranked SegNet and nnUNet, respectively. However, as seen in Figures 5C,E, there are conspicuous holes in the heart wall segmented by SegNet and nnUNet. Those results demonstrate that our S-Net can capture richer global context and local detail information than its counterparts (i.e., the other five CNN-based models). To verify that our S-Net can perform prediction more effectively with the Sigmoid activation function, we compared the results of training and testing using five other activation functions, namely LeakyReLU, Softmax, ReLU, PReLu, and Hyperbolic Tangent (Tanh). The results show that when the LeakyReLU, Softmax, and Tanh activation functions are used, the loss function (see Section 3.1.3) struggles to converge. The average DICE of the whole heart wall segmentation results are only 0.1348 and 0.2398, respectively, when ReLU and PReLU activation functions are used. In addition, it can be viewed from Table 2 that our S-Net is optimal for the segmentation of IA vasculature, except for the Specificity score, which is also attributed to the dual-branch cross-aggregation structure that effectively detects more complete small vessels.

**TABLE 1:** Quantitative comparison (mean ±95% confidence level) of different models for the whole heart wall segmentation regarding six evaluation metrics. The smaller the RVE, HD95, and ASSD values, the better the segmentation effect. The best results are highlighted in bold.

Models	DICE	Sensitivity	Specificity	RVE	HD95	ASSD
**3DUNet**	0.8018 ± 0.0271	0.8099 ± 0.0442	0.9918 ± 0.0024	0.0806 ± 0.0373	8.8262 ± 1.5267	2.4516 ± 0.3366
**SegNet**	0.7856 ± 0.0251	0.7914 ± 0.0400	0.9914 ± 0.0023	**0.0759 ± 0.0385**	9.1680 ± 1.4390	2.6365 ± 0.3613
**3DResUNet**	0.8297 ± 0.0253	0.8234 ± 0.0461	0.9935 ± 0.0026	0.0832 ± 0.0378	7.8312 ± 1.8458	2.1058 ± 0.3469
**KiU-Net**	0.8126 ± 0.0252	0.7899 ± 0.0416	0.9943 ± 0.0015	0.0905 ± 0.0442	7.7801 ± 0.9845	2.1789 ± 0.2700
**nnUNet**	0.8381 ± 0.0270	0.7770 ± 0.0426	**0.9971 ± 0.0010**	0.1485 ± 0.0497	9.1379 ± 2.0217	1.9339 ± 0.2751
**S-Net**	**0.9012 ± 0.0145**	**0.8947 ± 0.0388**	0.9966 ± 0.0010	0.0762 ± 0.0280	**4.6806 ± 1.7503**	**1.2233 ± 0.2146**

**TABLE 2:** Quantitative comparison of different models for IA vasculature segmentation.

Models	DICE	Sensitivity	Specificity	RVE	HD95	ASSD
**3DUNet**	0.8294 ± 0.0292	0.7437 ± 0.0477	0.9998 ± 0.0001	0.2098 ± 0.0566	48.3566 ± 15.4985	4.9263 ± 1.9197
**SegNet**	0.7081 ± 0.0345	0.5998 ± 0.0477	0.9995 ± 0.0001	0.3103 ± 0.0557	36.6319 ± 12.5793	4.8530 ± 1.1163
**3DResUNet**	0.8050 ± 0.1050	0.8173 ± 0.0655	0.9983 ± 0.0025	0.3467 ± 0.4517	48.2625 ± 14.2790	6.1627 ± 3.9610
**KiU-Net**	0.8213 ± 0.0273	0.7233 ± 0.0420	**0.9998 ± 0.0001**	0.2413 ± 0.0459	45.7190 ± 13.7946	4.7272 ± 1.2201
**nnUNet**	0.8342 ± 0.0501	0.7920 ± 0.0725	0.9995 ± 0.0003	0.1542 ± 0.0765	56.1059 ± 18.9685	6.6677 ± 2.9604
**S-Net**	**0.8735 ± 0.0288**	**0.9329 ± 0.0390**	0.9990 ± 0.0003	**0.1363 ± 0.0664**	**24.7471 ± 17.5222**	**2.2070 ± 1.0808**

The best results are shown in bold fonts.

### 4.4 Ablation studies

**FIGURE 7:**
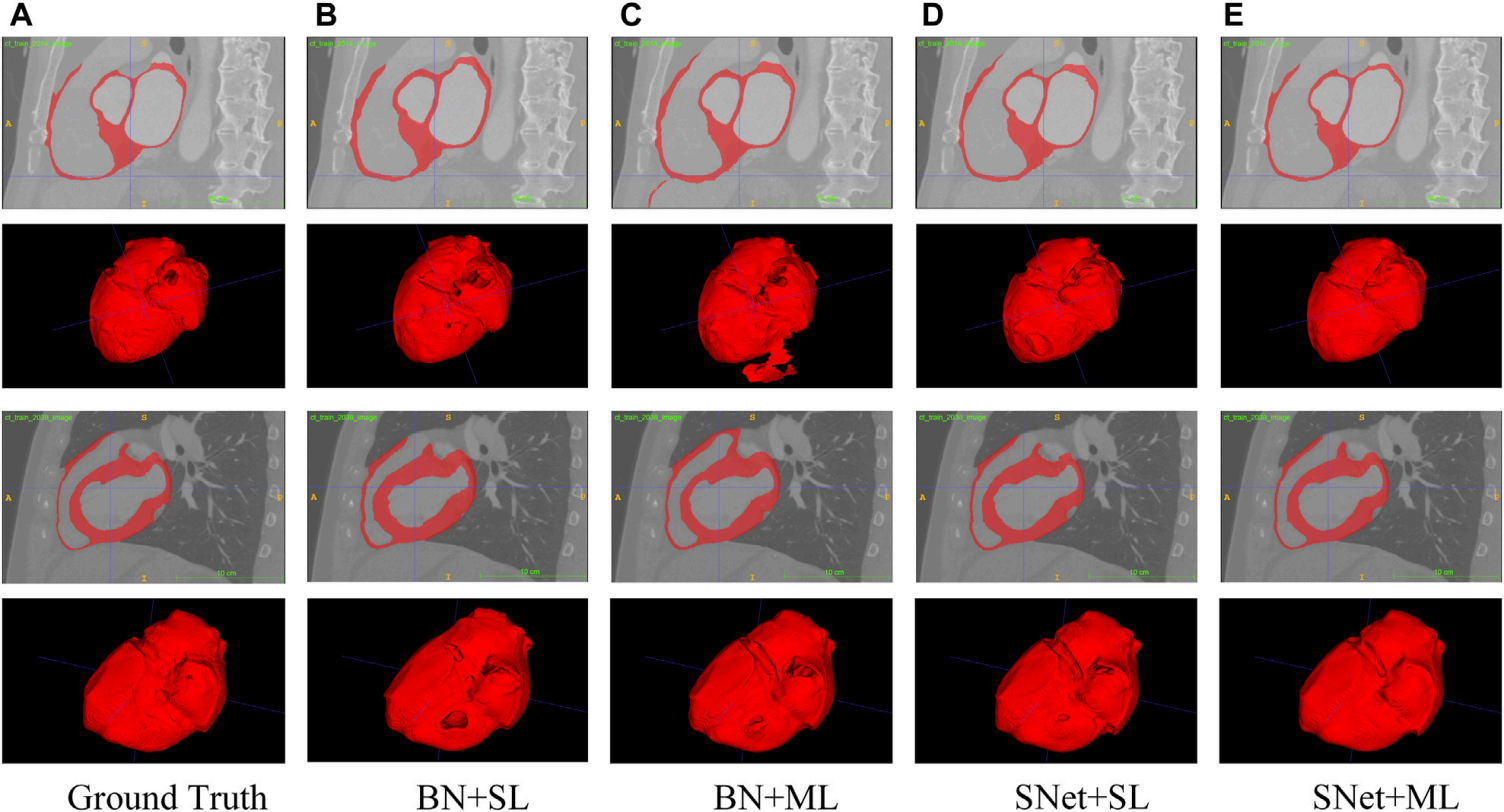
Visualization of the heart wall segmentation results generated by the four variants of the proposed S-Net. Images in column **(A)** are the Ground Truth and columns **(B–E)** represent the prediction results for baseline BN+SL, BN+ML, SNet+SL, and SNet+ML, respectively.

**FIGURE 8:**
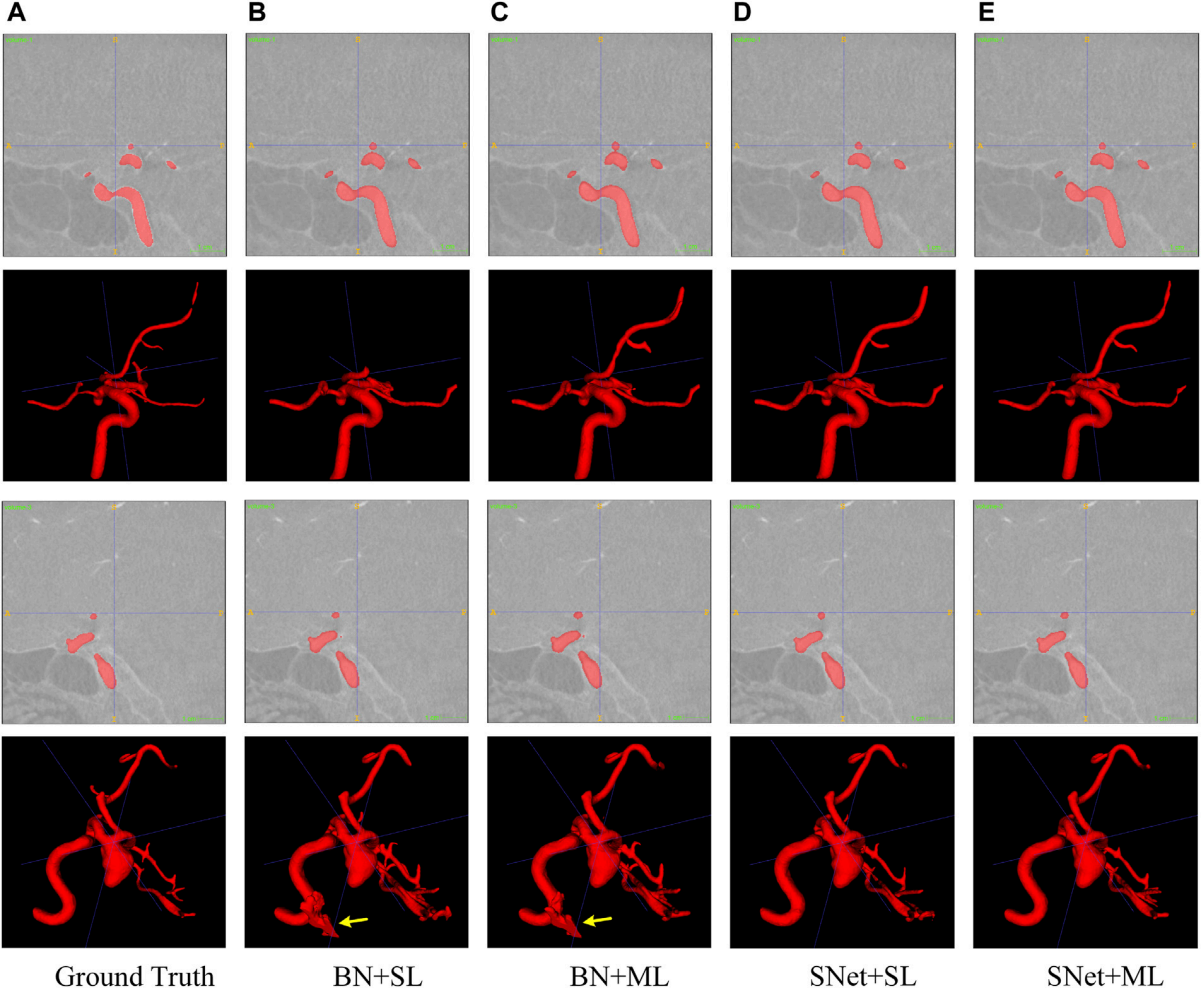
Visualization of the IA vasculature segmentation results generated by the four variants of the proposed S-Net. Images in column **(A)** are the Ground Truth and columns **(B–E)** represent the prediction results for baseline BN+SL, BN+ML, SNet+SL, and SNet+ML, respectively.

In this subsection, we perform a series of ablation experiments based on four variants of the proposed S-Net to validate the potential of our network architecture, including 1) a backbone network based on residual U-Net, which only uses the loss of a single decoding layer as supervision (denoted as BN + SL); 2) a backbone network with multiple decoding layer losses as supervision (denoted as BN + ML); 3) a dual-branch S-Net based on the loss of a single decoding layer (denoted as SNet + SL); 3) a dual branch S-Net utilizing multiple decoding layer loss (denoted as SNet + ML). All experiments were performed on the heart wall and IA vasculature datasets. Figures 7, 8 depict the subjective quality improvement of the configuration with a dual-branch encoder and a multi-supervised decoder. Tables 3 and 4 illustrate the objective results of these ablation experiments. We found that the performance improves as dual branch encoding and multi-supervised decoding are added to the network.

**TABLE 3:** Objective evaluation metric results of the four variants of the proposed S-Net for heart wall segmentation. The best results are highlighted by bold fonts.

Models	DICE	Sensitivity	Specificity	RVE	HD95	ASSD
**1) BN + SL**	0.8303 ± 0.0129	0.9286 ± 0.0208	0.9880 ± 0.0028	0.2381 ± 0.0594	6.8191 ± 0.5995	2.2405 ± 0.1257
**2) BN + ML**	0.8496 ± 0.0274	0.9147 ± 0.0262	0.9906 ± 0.0032	0.1586 ± 0.0956	25.7313 ± 18.5340	3.4594 ± 1.8512
**3) SNet + SL**	0.8836 ± 0.0121	0.9008 ± 0.0336	0.9947 ± 0.0013	0.0858 ± 0.0206	5.3262 ± 1.3749	1.4512 ± 0.1763
**4) SNet + ML**	**0.9012 ± 0.0145**	**0.8947 ± 0.0388**	**0.9966 ± 0.0010**	**0.0762 ± 0.0280**	**4.6806 ± 1.7503**	**1.2233 ± 0.2146**

The best results are shown in bold fonts.

**TABLE 4:** Objective evaluation metric results of the four variants of the proposed S-Net for IA vasculature segmentation. The best results are highlighted by bold fonts.

Models	DICE	Sensitivity	Specificity	RVE	HD95	ASSD
**1) BN + SL**	0.8025 ± 0.0575	0.9217 ± 0.0493	0.9979 ± 0.0012	0.3212 ± 0.2349	38.5696 ± 19.6079	4.9117 ± 3.4622
**2) BN + ML**	0.8103 ± 0.0319	0.9330 ± 0.0378	0.9981 ± 0.0005	0.3067 ± 0.1055	27.0335 ± 15.5266	2.8468 ± 0.9589
**3) SNet + SL**	0.8501 ± 0.0380	**0.9363 ± 0.0343**	0.9986 ± 0.0003	0.2055 ± 0.0569	**21.8773 ± 13.7142**	2.2607 ± 0.9994
**4) SNet + ML**	**0.8735 ± 0.0288**	0.9329 ± 0.0390	**0.9990 ± 0.0003**	**0.1363 ± 0.0664**	24.7471 ± 17.5222	**2.2070 ± 1.0808**

The best results are shown in bold fonts.

**Effectiveness of the dual-branch encoder.** From the results shown in Figures 7, 8, the two S-shaped structures (see d) and e)) are able to produce significant segmentation improvement compared to the two U-shaped baselines (see b) and c)). This implies that the crossed dual-stream encoding structure formed by adding the proposed resampling branch, attributed to the effective aggregation of global and local information, can remarkably optimize the overall segmentation performance. Specifically, the configuration of the dual-branch encoding effectively reduces the holes caused by inadequate heart wall segmentation (see b) vs. d) and c) vs. e) in Figure 7). Also, the dual-branch encoding avoids the noise (indicated by yellow arrows in Figure 8) generated by IA vasculature segmentation and achieves delineating more complete small vessels (see b) vs. d) and c) vs. e) in Figure 8). In addition, the performance of the objective evaluation metrics is also improved by introducing the dual-branch encoding; see 1) vs. 3) and 2) vs. 4) in Table 3, where the DICE scores are increased by 0.0533 and 0.0516, respectively, and similarly, see 1) vs. 3) and 2) vs. 4) in Table 4, where the DICE scores achieve gains of 0.0476 and 0.0632, respectively. These experimental results validate the necessity of the proposed dual-branch encoder.

**Effectiveness of multi-supervised decoder.** As shown in b) vs. c) and d) vs. e) of Figures 7, 8, replacing the single supervision (i.e., BN + SL or SNet + SL) in the decoder with multiple supervision (i.e., BN + ML or SNet + ML) also helps to improve the quality of heart wall and IA vasculature segmentation results. The improved results stem from the fact that the multi-supervision effectively integrates the high-level semantic and low-level localization knowledge, allowing the network to focus on the integrity of the target and the exactness of the details. This stipulation can also be confirmed by 1) vs. 2) and 3) vs. 4) in Table 3, where the DICE scores are improved by 0.0193 and 0.0176, respectively, and also, similarly, for 1) vs. 2) and 3) vs. 4) in Table 4, where the DICE scores gain 0.0078 and 0.0234, respectively. The results of these evaluation metrics presented in Tables 3 and 4 objectively demonstrate that the performance of the predictions is improved with the adoption of multi-supervision. Clearly, our comparison results show that the multi-supervised decoder optimizes the segmentation results to some extent.

## 5 Discussions

This study investigates the segmentation performance of the proposed network structure based on S-shaped multiple cross-aggregation for the whole heart wall and IA vasculature. The superior predictive ability of the proposed S-Net model is validated by comparison and ablation experiments, mostly outperforming the five compared state-of-the-art models in six evaluation metrics, where the DICE measured on two datasets (whole heart wall and IA) are 0.9012 and 0.8735, respectively. More importantly, qualitative experimental comparisons adequately demonstrate that our S-Net is capable of segmenting small/tiny structures, i.e., thin heart walls, e.g., the holes revealed by other segmentation models (indicated by the arrows in Figures 5B–F), and tiny arteries (indicated by the arrows in a) and g) of Figure 6).

It is clear from the experimental results that the proposed S-Net is a good backbone architecture for small-volume segmentation. Three key strategies attributed to our success. First, we design an efficient two-branch encoder, i.e., a regular downsampling encoding branch with progressively halved resolution network layers and a resampling encoding branch with fixed resolution network layers, to explore spatial details and global contextual information. This configuration allows the encoder to consider large and small receptive fields, which can efficiently guide the CNN model to capture small/thin structures and fine-grained details. Our intuitive explanation of the abovementioned dual-branch encoder can be seen in Figures 7, 8. Second, our multiple cross-aggregation module also plays a vital role in guaranteeing the comprehensiveness and robustness of encoded features. In other words, the module effectively integrates multilayer high-level and low-level features. Since high-level features contain rich semantic and global context knowledge, while low-level features have plenty of details and localization information, the propagation of the fused features to the decoder through the hierarchical horizontal connection strengthens the global dependency and local details of the decoded features, facilitating the detection of small volumes. Third, we perform multilevel supervised prediction for all decoding layers, effectively combining high-level and low-level features with different semantics and resolutions along a top-down path, optimizing the network’s learning efficiency for targets of various sizes and thus improving the completeness of prediction for tiny structures. The improvement in segmentation performance by the multi-supervised decoder can be seen in Tables 3 and 4. Collectively, the three proposed strategies allow our model to achieve state-of-the-art performance and have the ability to segment small volumes effectively.

Besides cardiovascular applications, the proposed S-Net could also be applied to segment vasculature in other organs, e.g., hepatic veins/arteries and retinal vessels. We also stipulate that the proposed S-Net could also be used in oncological applications (e.g., brain tumors, colon cancer, breast cancer, lung nodules). For instance, the improved detection of small targets allows us to identify small, hard-to-detect tumors and complex tumor compositions. As a result, the proposed S-Net can be integrated into an artificial intelligence (AI) system to enhance the prediction and detection of disease progression, thereby elevating the clinical management of cancer patients. It is worth noting successes of such predictive modeling have been achieved in predicting the growth of abdominal aortic aneurysms (Rezaeitaleshmahalleh et al., 2023a; Rezaeitaleshmahalleh et al., 2023c) and the rupture status of intracranial aneurysms (Sunderland et al., 2021; Jiang et al., 2023). In the future, we will expand the application of the proposed S-Net to oncological applications.

Although our two-branch coding structure facilitates the extraction of detailed features, it has the drawbacks of high computational complexity and long training time, and its training process requires high-performance hardware with large amounts of memory and is time-consuming. Our future work will optimize the training model by reducing the parameters through regularization and model pruning. Furthermore, the training time will be reduced by performing batch normalization and adding pooling layers to allow fast convergence.

## 6 Conclusion

In this paper, motivated to overcome the drawbacks of existing U-shaped segmentation architectures, we propose an S-Net framework for small/thin structure segmentation of medical images. Our novelty lies in exploring a dual-branch encoder consisting of resampling and downsampling convolutional layers to capture the information from large and small receptive fields for more accurate learning of small targets and finer details. These two branches are efficiently integrated by leveraging a novel S-shaped multiple cross-aggregation approach for effective training. Meanwhile, we enhance the global context and local detail knowledge in the decoding stage by propagating complementary features from the encoding layers through lateral connections. We also supervise features from all decoding layers in the top-down path to fully optimize the semantics and localization of the prediction results. To verify the superiority of the proposed S-Net for small/thin structure predictions, we performed segmentation experiments on the heart wall and IA vasculature, and the results demonstrated that the proposed model outperforms state-of-the-art CNN methods, significantly improving the structural accuracy and surface quality of segmented volumes and having the ability to adequately capture small structures, which possesses the potential to facilitate clinical applications.

## Data Availability

The datasets presented in this study can be found in online repositories. The names of the repository/repositories and accession number(s) can be found below: https://zmiclab.github.io/zxh/0/mmwhs/ and http://ecm2.mathcs.emory.edu/aneuriskweb/about.

## Footnotes

1 Centers for Disease Control and Prevention, National Center for Health Statistics (NCHS). About Multiple Cause of Death, 1999–2020. CDC WONDER Online Database website. Atlanta, GA: Centers for Disease Control and Prevention; 2022. Accessed February 21, 2022.

2 For details of the MM-WHS dataset, please refer to https://zmiclab.github.io/zxh/0/mmwhs/data.html
